# M. Huguier on Croup in the Adult

**Published:** 1842-05-07

**Authors:** 


*Croup in the Adult.*—At the Royal Academy of Medicine, March 8th, M. Huguier presented the larynx, &c. of a female who had died of croup. The woman was 24 years of age, and the disease was unaccompanied by the cough peculiar to croup; the only symptoms present were aphonia and the hissing respiratory sound. The patient died suddenly in forty hours from the commencement of the attack, without any signs of asphyxia, suffocation, or lividity of the countenance.

On examination, after death, false membranes were found lining the amygdalæ, the pharynx, larynx, trachea, and upper divisions of the bronchi. The right cavities of the heart contained fibrous clots, which adhered firmly to the walls of the heart, and sent off various prolongations between the carneæ columnæ and into the pulmonary artery. The author seems inclined to attribute the sudden death of the patient to the coagulation of blood in the heart.—*Ibid.* March 19th, 1842.

